# The Relationship between Different Types of Alarm Sounds and Children’s Perceived Risk Based on Their Physiological Responses

**DOI:** 10.3390/ijerph16245091

**Published:** 2019-12-13

**Authors:** Jiaxu Zhou, Xiaohu Jia, Guoqiang Xu, Junhan Jia, Rihan Hai, Chongsen Gao, Shuo Zhang

**Affiliations:** 1 Architecture College, Inner Mongolia University of Technology (IMUT), Hohhot 010051, China; xgq5@imut.edu.cn (G.X.); hairihan1012@126.com (R.H.); gaocso@163.com (C.G.); 2 Inner Mongolia Engineering Technology and Research Center for Green Buildings, Architecture College, Inner Mongolia University of Technology, Hohhot 010051, China; 3 Inner Mongolia Key Laboratory of Green Building, Architecture College, Inner Mongolia University of Technology, Hohhot 010051, China; 4 Beijing Aidi School, Beijing 100000, China; junhanjia@126.com; 5 UCL Institute for Environmental Design and Engineering, The Bartlett, University College London (UCL), London WC1H0NN, UK; ucbqsz0@ucl.ac.uk

**Keywords:** children safety, perceived risk, alarm sound, physiological monitoring, electrodermal activity, heart rate variability

## Abstract

Due to differences in cognitive ability and physiological development, the evacuation characteristics of children are different from those of adults. This study proposes a novel method of using wearable sensors to collect data (e.g., electrodermal activity, EDA; heart rate variability, HRV) on children’s physiological responses, and to continuously and quantitatively evaluate the effects of different types of alarm sounds during the evacuation of children. In order to determine the optimum alarm for children, an on-site experiment was conducted in a kindergarten to collect physiological data for responses to different types of alarm sounds during the evacuation of 42 children of different ages. The results showed that: (1) The alarm sounds led to changes in physiological indicators of children aged 3–6 years, and the effects of different types of alarm sounds on EDA and HRV activities were significantly different (*p* < 0.05). Skin conductance (SC), skin conductance tonic (SCT) and skin conductance level (SCL) can be used as the main indicators for analysing EDA of children in this experiment (*p* < 0.05), and the indicators of ultralow frequency (ULF) and very low frequency (VLF) for HRV were not affected by the type of alarm sounds (*p* > 0.05). (2) Unlike adults, kindergarten children were more susceptible to the warning siren. The combined voice and warning alarm had optimal effects in stimulating children to perceive risk. (3) For children aged 3–6 years, gender had a significant impact on children’s reception to evacuation sound signals (*p* < 0.05): Girls are more sensitive than boys in receiving evacuation sound signals, similar to findings of studies of risk perception of adult males and females. In addition, the higher the age, the greater the sensitivity to evacuation sound signals, which accords with results of previous studies on the evacuation dynamics of children.

## 1. Introduction

Although researchers have long studied the behaviours of human evacuation, it is still a complex scientific problem to understand the evacuation behaviours of children. Children are vulnerable groups during fires [1]. In China, there are now more than 220 million children between the ages of 0 and 14, but research on evacuation of children in emergencies is rare. The cognitive and motor abilities of children are significantly different from those of adults [2]. The problem of child evacuation lies in their inability to fully perceive and respond to risks [3,4,5]. Therefore, understanding how children perceive the risks they face is critical to safety research on preschool educational buildings.

### 1.1. Evacuation Behaviours

Various evacuation simulation models have been developed based on evacuee behaviour in previous studies of evacuation issues. These models can be generally classified into the cellular automaton model (CA), the lattice gas model (LG), the social force model (SF), and the agent-based model (AB). A number of commercial tools have also been developed to simulate the behaviour of evacuees, such as Pathfinder [6] and EXODUS [7]. These evacuation models and simulation tools are widely used in fire control training, evacuation drills and education, and to some extent, can explain and predict the behaviour of the evacuees, and the entire evacuation process. However, due to the excessive simplification of the behaviour patterns of the evacuees and the lack of legitimacy of the various assumptions about evacuation behaviours, most of these models and tools cannot provide accurate simulations and predictions of evacuation behaviours or overall evacuation processes. Of these, there are few evacuation models for children. The child evacuation status must be fully understood before a model of child evacuation can be developed [8]. 

In recent years, the study of the evacuation of children has attracted the attention of researchers. Some countries have collected some data on the evacuation of children, mainly focusing on children’s speed of movement (horizontal or by stairs), flow-density relationships, (pre)evacuation time and their route selection [2,3,5,9,10,11,12,13]. How to evaluate children’s own perception of risk has not been covered in previous studies, leading to slow progress in research on children’s evacuation behaviour and subsequent development of simulation models.

### 1.2. Alarm Sounds

Sound has proven to be an important medium to motivate occupants to evacuate buildings with reduced evacuation times [14]. Previous experiments on adult evacuation showed that recorded voice alert messages were more effective than warning alarms in motivating occupants to evacuate buildings. Purser, in their 2001, 2010 studies, monitored evacuations in a shopping centre, a theatre, a large office building and a university teaching room under hidden video surveillance, in order to examine responsiveness to different alarm systems (including a sounder and long and short voice messages) and provide pre-movement time data [15,16]. The findings from both the monitored evacuations and the experimental study were that voice alarms provided more reliable and shorter pre-movement response times than sounders. British researchers have reached the same conclusions when testing different alarm systems in various buildings such as shops, stadiums, exhibition halls, and leisure centres in London [17]. It was worth noting that earlier studies had found similar results [18,19,20]. In the past, most of the research objects have been buildings such as residences, hospitals, and transportation hubs. There has been no research on the effects of different types of alarm sounds on children’s evacuation in public spaces like kindergartens, that are crowded with children. The subjects of previous research were all adults, and there has been no experimental research on the evacuation of children led by alarm sounds. If a more scientific and objective measurement method (physiological indicators) can be used to identify which types of alarm sounds are better for their risk perception, this would be very beneficial for children’s evacuation and safety.

### 1.3. Risk Perception of Evacuees

Considering that human evacuation behaviour is a conscious response to emergencies and risks, risk perception is a key step in triggering human evacuation behaviour. The most widely used method for research of evacuee behaviour and risk perception has been to conduct post-surveys of survivors of a fire, including questionnaires or interviews [21,22]. Tong and Canter have described how humans deal with dangerous situations in three main steps, including detecting signals of danger, perceiving and understanding risks, and predicting the consequences associated with decision options [23]. It was worth noting that lengthy self-report questionnaires and interviews are not appropriate for young children [1,3]. Meanwhile, there has been research that has reproduced emergency and evacuation processes using data analysis based on surveillance videos and official incident reports [24]. This approach allows for reasonable behavioural analyses based on actual data but is limited by the scarcity and imperfections of such data, due to the lack of current records and poor usability [25]. In addition, some researchers have used virtual reality (VR) environments to study evacuation behaviour [26,27], but this approach requires extensive guidance before the experiment to familiarise the participants with the process, which is too complicated for children aged 3–6, and therefore inapplicable. Therefore, there is an increasing demand for an objective and non-invasive approach that can continuously evaluate the risk perception of children to explore the mechanism behind their behaviour.

### 1.4. Physiological Responses

The existing research indicated that sound had a significant effect on the function of the human nervous system [28,29,30,31]. The human nervous system is divided into the Central Nervous System (CNS) and the Peripheral Nervous System (PNS). The PNS includes the somatic nervous system (SNS) and the autonomic nervous system (ANS). The ANS consists of sympathetic nerves and parasympathetic nerves. Sympathetic nerves transmit excitatory signals activating the body [32]. When faced with stressful conditions, sympathetic nerves are activated to generate the “fight or flight” response. When stressful conditions are over, the parasympathetic nerves act to restore balance. Studies have shown that these responses can be expressed by indicators of electrodermal activity (EDA) and heart rate variability (HRV). For example, skin conductivity (SC) was positively correlated with the degree of emotional arousal [33]. Sympathetic excitation caused by risks dominates the heart’s nervous activity, causing changes in physiological responses associated with cardiac activity. The inter-beat interval (IBI) in HRV was related to risk perception [34], and low frequency (LF) and high frequency (HF) reflect the activity of the sympathetic and parasympathetic nervous systems, respectively [35]. At the same time, methods for monitoring physiological activity have been widely used in various studies related to human health, perception, and emotion [36].

EDA and HRV can be used to evaluate risk perception because the sympathetic excitation caused by human perception of risks leads to substantial changes in EDA and HRV. In addition, mobile physiological sensors are an emerging tool. Recent advances in wearable technology have enabled us to overcome the hardware limitations of traditional EDA and HRV sensors to better understand the effects of experimental variables on physiological responses in real-world environments. Studies have applied wearable sensors to research related to risk perception, human health, and psychology. For example, one study collected EDA data from construction workers to study their risk perception of high-risk construction operations [37]. Despite its potential, the feasibility of using EDA and HRV data obtained through wearable sensors to understand the risk perception in the evacuation of children has not been well studied.

### 1.5. Aims and Contributions

In order to fill this gap, this study collected physiological data through field experiments to understand the state of children during an evacuation. The authors proposed a new method for continuously and quantitatively evaluating the risk perception of children based on physiological data (e.g., heart rate and electrodermal activity) obtained by wearable sensors. In this context, the aim of this study was to investigate the physiological responses of children to different types of alarm sounds in emergencies, in order to evaluate the influence of the type of alarm sounds on the perceived risk to children. Specifically, this article focuses on the following:

1. What is the significance of the effects of different types of alarm sounds on the physiological indicators of children?
2. What are the effects of three different types of alarm sounds (voice alert, warning alarm and combined) on the physiological responses of children, and which type of alarm sound is more effective in alerting children to perceive risks?
3. Does age/gender have an influence on children’s reception of alarm sound signals to perceive risks?

The main contribution of this study is demonstrating the feasibility of using multi-channel wearable sensors to evaluate the risk perception of children in emergencies, continuously. Given the complexity and dynamics of the evacuation of children, the use of an objective, continuous, non-invasive method of monitoring children’s physiological responses will help to develop a deeper understanding of the evacuation behaviour of children.

## 2. Methods

In this study, wearable sensors were used to monitor the physiological indicators of children. Three typical types of alarm sounds were randomly presented to the experimental participants. The experimental process was recorded by a camera for behavioural observation, and the obtained data were analysed statistically.

### 2.1. Experimental Setting

A typical medium-sized city, Hohhot in Inner Mongolia, was selected, and Hua Di Kindergarten was selected for the experimental site after research on city architecture because it is a city kindergarten of common size. The kindergarten was a building with three floors. Children were classified into the “junior” (3–4 years old), “middle” (4–5 years old), and “senior” classes (5–6 years old) [3,5,10]. The experiment took place in the classroom used for children’s daily activities, and cameras were placed at the corners of the classrooms to record the entire experimental process.

### 2.2. Participants

In this study, EDA data were obtained from 42 kindergarten children (14 from the senior class, 14 from the middle class and 14 from the junior class), which are shown in detail in Table 1. The data collection agreement was approved by Hua Di Kindergarten and the parents of the children, who also signed the informed consent form. Meanwhile, ethical approval for the study was provided by the Inner Mongolia University of Technology, Architecture College Ethics Committee. All subjects had normal hearing and were not taking any psychotropic medications, and no clinical condition was reported (e.g., cardiovascular disease) that might have affected their physical and mental ability to perform daily tasks. Considering the daily routine of the kindergarten (children would have noon break and get up at 2:00 p.m.), the experiment was arranged at 3:00 p.m., so subjects would not have experienced strenuous exercise or fatigue within two hours before the experiment.

### 2.3. Physiological Measurements 

**Figure 1:**
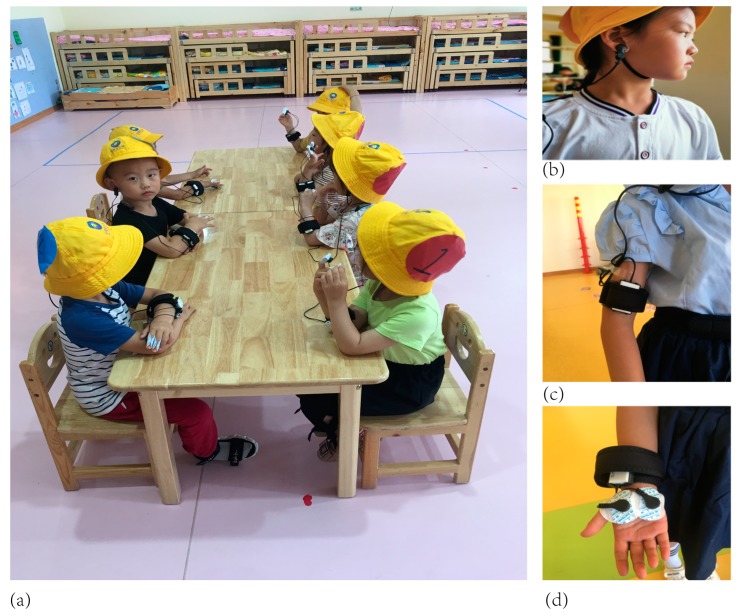
(**a**) Experimental scene; (**b**,**c**) Data collector and receiver for heart rate variability (HRV); (**d**) Data collector and receiver for electrodermal activity (EDA).

In this experiment, the EDA and HRV data were collected during the evacuation of children as the main means of analysing physiological indicators. The collection of physiological data was carried out as follows:

(a) EDA: The conductivity of the skin surface was measured by a wireless electrodermal sensor. The two electrodes were connected to the fingertips or the palm of the hand (Figure 1). Changes in physiological characteristics could be measured by the conductivity value. In terms of EDA, the SC score can be used to describe the conductivity of the skin, and can be calculated using the scores of skin conductance tonic (SCT), skin conductance phasic (SCP); the SC score is equal to the SCT score plus the SCP score (SC = SCT + SCP). The EDA values were captured in the experiment using a multi-channel physiological recorder. Specific indicators are as follows: SC score, SCT score, SCP score, skin conductance level (SCL), amplitude (AMP) of all event-related skin conductance responses (SCR) and response latency for each event stimulus (LATENCY).

(b) HRV was measured using wireless photoplethysmography (PPG). The principle of measurement is that when the light beam of a certain wavelength irradiates onto the skin surface, the light beam will be transmitted to the photoelectric sensor through transmission. When the heart contracts, the absorption of light is the strongest, and the detected light signal intensity is the weakest. When the heart is dilated, the detected light signal intensity is the strongest. Thus, the light signal detected by the photoelectric sensor exhibits changes fluctuating with the heartbeat. The ear clip of the pulse sensor should be clipped to the ear, and the sensor should be attached to the wrist with a wrist strap (Figure 1c,d). The HRV was calculated from the IBI [34]. The standard deviation of all NN intervals (SDNN), The NN interval is a way of saying the cardiac time interval between peaks, the square root of the mean of the sum of the squares of differences between adjacent NN intervals (RMSSD), and short-term measures of the frequency domain, including ultralow frequency (ULF) power (0–0.0033 Hz), very low frequency (VLF) power (0.0033–0.04 Hz), LF power (0.04–0.15 Hz), HF power (0.15–0.4 Hz) and LF/HF, were tested in this study.

The above physiological indicators have been widely used in research on risk perception and health in recent years, and have been shown to be able to sensitively reflect the physiological changes of individuals under stress. Because the alarm sound can be seen as a stress stimulus causing stress in the body or changes in the arousal level of the listener [38,39], and both EDA and heart rate are physiological indicators that rapidly respond to physiological arousal and stress stimuli [40,41,42], the mean SC score and heart rate would increase significantly under virtual stress stimulation, which are effective indicators of physiological response under stress stimulation [43]. SCL and SCR are also used to measure risk perception [44]. In addition, the above indicators of EDA have been widely used in relevant research of children [45,46] and effectively reflect physiological changes in children; therefore, they are also applicable to this study based on children’s evacuation behaviour.

### 2.4. Experimental Procedure

The evacuation experiment was semi-announced [3,11,12]; that is to say, the teachers knew about it, but the children did not. In order to ensure that the children participating in the experiment did not know what was ahead, the teachers told them they were going to play games when we installed the sensors on the children. There were no distracting objects in the room other than the experimental equipment required [47,48].

**Figure 2:**
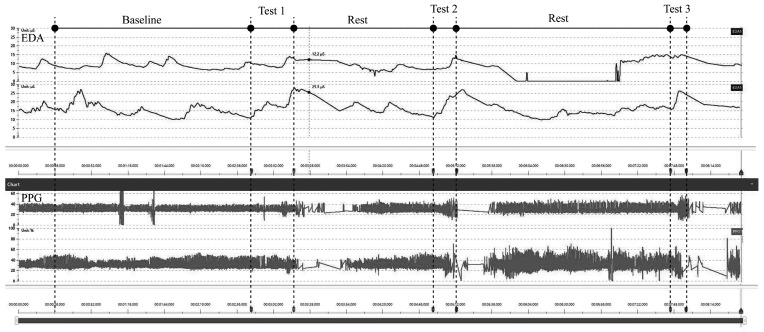
Interface of the experiment data collection platform.

First, the children were asked to sit in chairs. The research team helped them put on the sensors (Electrodermal Activity, EDA; Photoplethysmography, PPG) and distributed the numbered hats to the children. Boys were assigned blue numbers and girls red numbers (Figure 1). The ERGOLAB platform (Kingfar International, Beijing) was used to collect their physiological data. The recording process of physiological signals is shown in Figure 2. The sensors and each subject’s skin were sterilised in advance to eliminate any dirt that might hinder the function of sensor electrodes. The research team then checked whether the sensors were in place, and turned on the sensors to pair with the computer for signal reception.

Baseline measurements of the children’s physiological data were performed two minutes prior to the start of the experiment, and the physiological signals were calibrated. After that, formal experiments were started to record physiological data during child evacuation. We randomly presented the same three types of typical alarm sounds to different groups. The descriptions of the three sound types are shown in Table 2. The alarm sound was played by Bluetooth audio in the activity room. When the alarm sounds went off, the experiment began, and the camera was turned on at the same time, and when the children were evacuated from the activity room, the experiment ended.

Alvarsson suggested a half-life of SCL of 120 s [49]. Therefore, in order to avoid the interference of physiological indicators between two adjacent experiments, 10 min were given for rest between experiments [27,31]. Experiments of different groups of children took place in their respective classrooms, which were not on the same floor, so they would not communicate or interfere with each other. In addition, all subjects’ activities were recorded using a camera, which allowed synchronous analysis of their behaviour when further analysing the relationship between physiological data, perceived risk and experimental variables.

### 2.5. Data Analysis

Of the 42 subjects, one had to go to a Taekwondo class and failed to complete all the experiments due to time conflicts, so the data of a total of 41 subjects were collected. A database containing the final results was created using IBM SPSS 25.0 [27,31,50,51] to evaluate the effect of different types of alarm sounds on the risk perception of the children in the evacuation experiments.

The EDA indicators (SC, SCT, SCP, SCL, AMP, LATENCY) and HRV indicators (IBI, SDNN, RMSSD, ULF, VLF, LF, HF, LF/HF) of all subjects at baseline, and after exposure to the three types of alarm sounds were recorded. Data analysis was performed using the following methods:

- By repeating the measurement, based on paired *t*-tests 95% confidence level, the statistical significance of the differences between the scores was further evaluated to determine the differences among the physiological data corresponding to different types of alarm sounds.
- Normalisation was performed of the baseline data of all data points relative to the resting state and all individual differences between subjects were eliminated, so that the processed data could be compared to the results obtained without alarm sound stimulation.

## 3. Results

### 3.1. The Significance of the Influence of Alarm Sound on Different Physiological Indicators

#### 3.1.1. Electrodermal Activity, EDA

Table 3 shows the EDA scores at baseline and after exposure to the three types of alarm sounds. It can be seen that the values of EDA indicators after exposure to alarm sound intervention were significantly higher than the baseline values, suggesting that the intervention of the alarm sounds caused increased EDA of the children. The SC, SCT and SCL scores were significantly higher after exposure to S2 and S3 than to S1, which constituted the basis of subsequent analysis. For further verification, we performed a paired *t*-test on the results, as seen in Table 4. We use the *p*-value of 0.05 to distinguish the level of significance [27,31,34]. The results of the *t*-test showed that alarm sounds had a significant influence on the three physiological indicators SC, SCT and SCL (*p* < 0.05) of children, which was basically consistent with the results of studies on EDA indicators and risk perception in adults [27,37,44]. Monitoring the SC, SCT and SCL scores of the EDA indicator could be used to analyse whether different types of alarm sound stimuli activated children’s risk perception.

Meanwhile, the results of the paired *t*-test showed that when children were exposed to different types of alarm sounds (S1, S2 and S3), values of the same indicators were also significantly different between groups. This means that the above three indicators can be used to distinguish the degree of physiological changes of subjects after exposure to different types of alarm sounds.

It is worth noting that there were no statistically significant pairwise differences in the LATENCY and AMP scores representing EDA among the baseline data and data after exposure to the three types of alarm sounds (*p* > 0.05). This means that LATENCY and AMP reflect the transient rapid activity of EDA in sound intervention experiments and may be affected by various factors irrelevant to experimental control (e.g., finger movement), and SCP (*p* > 0.05) may also be affected by finger movement. Therefore, they were not used as the main indicators in the subsequent analysis.

#### 3.1.2. Heart Rate Variability, HRV

The scores of HRV measurements for all subjects are summarised in Table 3. As can be seen from the table, when the subjects were exposed to the alarm sounds S1, S2 and S3, the LF/HF value decreased, and the other indicators increased compared to the baseline data. This means that the intervention of the alarm sounds caused changes in the HRV of children.

Paired *t*-tests were also performed to further evaluate the statistical significance of the differences in the scores of HRV indicators (Table 4). The results showed that there were significant differences in HRV indicators between the baseline data and the experimental data (*p* < 0.05), especially in LF and HF (*p* < 0.05), except for ULF and VLF (*p* > 0.05), suggesting that the differences between groups were statistically significant, and the children showed significant changes in HRV indicators when exposed to alarm sounds. Monitoring the HRV IBI, LF, HF and LF/HF scores of the HRV indicator could be used to analyse whether different types of alarm sound stimuli activate children’s risk perception differently.

The paired *t*-test results showed that when children were exposed to different types of alarm sounds (S1, S2 and S3), the values of the HRV indicators were significantly different (*p* < 0.05), and values of the same indicators were also significantly different between groups. However, this only partially held for ULF and VLF, which thus, did not suffice to distinguish children’s HRV conditions. Therefore, ULF and VLF were not important indicators for the subsequent analysis of the physiological changes of children with different types of alarm sounds.

### 3.2. Effects of the Alarm Sound Types on Children’s Perceived Risk

**Figure 3:**
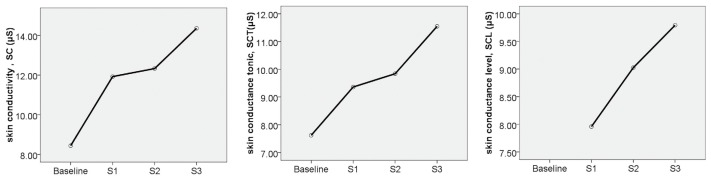
Changes in the mean value of EDA indicators by sound type.

**Figure 4:**
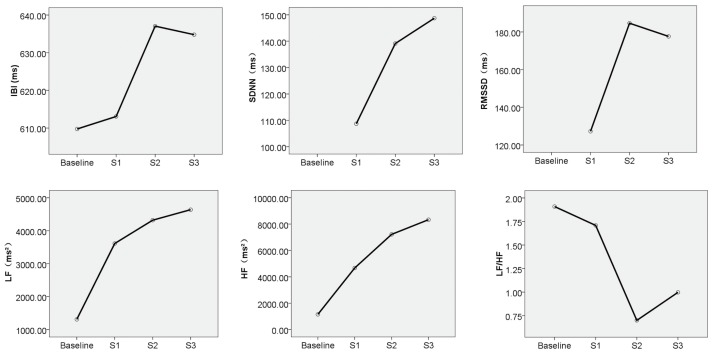
Changes in the mean values of HRV indicators by sound type.

According to the paired *t*-test results described in the previous section, three physiological indicators for analysing EDA were used: SC, SCT, and SCL; similarly, IBI, SDNN, RMSSD, LF, HF, and LF/HF were considered indicators for analysing HRV. The means of all physiological indicators after exposure to different types of alarm sounds were calculated (Table 3). Using the data presented in Figure 3 and Figure 4, the effects of the different types of alarm sounds on each physiological indicator were determined.

Figure 3 presents the changes in the mean values of the EDA indicators for different sound types. In terms of SC, the lowest SC value was obtained for the voice alert (S1). While the effect of warning alarm (S2) was slightly stronger, its SC value was not significantly different from the baseline value. The combined voice alert and warning alarm (S3) had the highest SC value. This means that the EDA indicators of children receiving S3 were significantly higher than those receiving S1 and S2. Similarly, it can be seen from the SCT changes that the variation trends of SCT and SC in different experimental scenarios were the same, and S3 had the highest SCT score, followed by S2 and S1. However, SCT changes in children receiving S3 were more pronounced than SC changes. SCL is also an important indicator of the EDA level. S3 had the highest SCL score, S1 had the lowest SCL score, and the SCL curve showed greater amplitude of variation than the previous two indicators.

From the above analysis, it was found that if S2 and S3 were classified into the warning alarm category, and S1 was classified into the language category, and significant differences can be observed between the two. Therefore, the warning alarm can increase the values of SC, SCT, and SCL, which means that the warning alarm signal can better activate the sympathetic system, leading to the alertness of the children, and promote secretion by the sweat glands to improve the skin conduction level. S3 had the most significant effect.

As shown in Figure 4, from the results of time-domain measurement, the IBI values under S2 and S3 were significantly different from the baseline value, while S1 did not show such a significant effect as S2 and S3, suggesting that a warning alarm is better than a pure voice alert. The order of the SDNN values of the three alarm sound types was S3 > S2 > S1, which means that the combined voice alert and warning alarm (S3) triggered a more significant sympathetic activation in the children, causing a more significant HRV response than the other two types of alarm sounds, and making it easier for children to perceive risks. In terms of RMSSD, S2 and S3 scored higher than S1, and the difference between S3 and S2 was not obvious.

According to the results of frequency-domain measurements, the LF value was significantly higher than the baseline value under all three types of alarm sounds. Specifically, S1 had the lowest LF value, followed by S2 and S3, which was the same as in the case of the SDNN indicator. From the change in HF values, the same variation trends in LF values could be seen in HF values among the three sound types, and S3 has the greatest effect on children. LF/HF is also an important indicator in HRV analysis, representing the equilibrium state of sympathetic and parasympathetic tone. The results show that all three sound types could break the balance of sympathetic and parasympathetic tone, but the effect of S1 on breaking the equilibrium state was not as obvious as with S3 and S2.

Among the HRV indicators, the three sound types can cause IBI, SDNN, RMSSD, and LF to increase, and the effect of S3 was the most significant. The sounds (S1, S2 and S3) that lead to negative psychological reactions such as anxiety, stress and unpleasantness, led to a significant decrease in HR and LF/HF, which is consistent with previous studies [27,31,52,53,54,55,56,57]. Moreover, the experimental results show that the warning alarm could better activate the risk perception of children than a pure voice alert, enabling them to evacuate more quickly, and shortening the evacuation pre-action time. In particular, S3 showed the best results, holding the greatest potential to improve evacuation efficiency.

In summary, children were more sensitive to the warning alarm, which is different from the results of previous studies of evacuation alarms in adults. Adults are more sensitive to voice alerts, and such voice messages should be complete sentence instead of short keywords [15,16,58]. One likely reason for this difference is that children aged 3–6 years have significantly different cognitive abilities from adults, having not developed good feedback to language. In other words, long voice messages are difficult for children to understand.

### 3.3. Effects of Gender/Age on Children’s Risk Perception

Considering S2 was the existing alarm sound in the fire protection system of Huadi kindergarten, and according to the results of the previous section, the existing warning alarm showed better effects than voice alert. Therefore, this section analyses and discusses the effects of gender and age on children’s risk perception based on the data obtained under the S2 experimental condition.

#### 3.3.1. Gender

For children aged 3–6, does gender affect the children’s sensitivity to evacuation sound signals? To explore this issue, all subjects under the S2 experimental condition were gender-differentiated, and a paired *t*-test at a 95% confidence level was performed.

**Figure 5:**
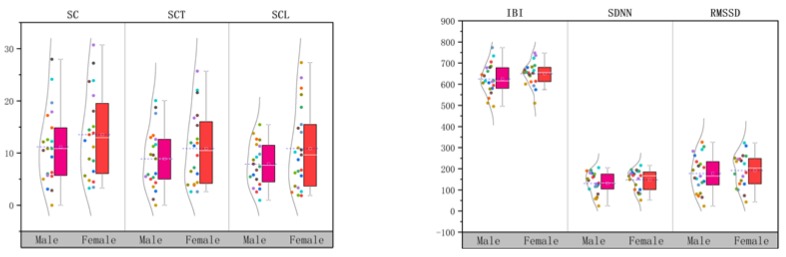
EDA and HRV by gender in the test of S2.

The test results showed that except for SCP, the other physiological indicators were significantly affected by gender (*p* < 0.05), indicating that gender significantly affected the risk perception of children. The scores for physiological indicators at baseline and under S2 are summarised in Table 5 based on gender, and the data are visualised in Figure 5. The experimental data for each physiological indicator at baseline and during the experiment were analysed, and the relative change was calculated by Formula (1) [31], so as to normalise all data points relative to the baseline to remove individual differences between subjects, and the processed data could then be compared to the results obtained without an alarm sound stimulus.

*Relative change (%) = ((alarm sounds test value − baseline value)/baseline value) × 100* (1)

Among EDA indicators, SC and SCT scores were significantly higher in boys and girls during the S2 test than at baseline (Table 5, Figure 5), and the relative changes of the scores in girls were higher than in boys, with a relative change of SC 18.33% greater than that of SCT, 15.27% greater in girls than in boys, indicating that girls’ skin conductivity was higher under the action of S2. In other words, from the perspective of EDA, girls are more sensitive than boys to the perception of risk when receiving evacuation sound signals.

Similarly, the values of HRV indicators of the subjects during the S2 experiment were also significantly higher than those at baseline, indicating that the sympathetic activation induced by the children’s perceived risk caused a substantial change in EDA and HRV (Table 5, Figure 5). Girls had a higher relative change than boys. Specifically, the analysis of the relative change of IBI, SDNN, RMSSD, LF, HF and LF/HF from the baseline to the S2 experiment was performed after grouping the 41 subjects by gender. According to the analysis, the relative changes in all seven physiological indicators for HRV were lower in boys than in girls. Among them, IBI, SDNN, RMSSD, LF and HF increased from the baseline to the S2 experiment, but boys had lower scores than girls, and the relative change was positive. LF/HF showed a downward trend from the baseline period to the S2 experiment, the relative change was negative, and boys had higher scores on these two indicators than girls did. In summary, the amplitude of variation of HRV indicators was lower in boys than in girls, indicating that girls are more sensitive than boys to alarm sound signals.

The gender-disparity analysis shows that girls give greater attention to risk and maintain a higher degree of vigilance when they are exposed to danger signals. This result is consistent with the findings of studies of risk perception in adults by Rundmo and Drottz [59,60]. However, previous studies used questionnaires and interviews, which are relatively cumbersome and difficult for children aged 3–6. This study, from an objective point of view, provides measures for research on the effects of gender on risk perception of children based on changes in physiological indicators.

#### 3.3.2. Age

In order to investigate whether age has an impact on the risk perception of children when receiving an evacuation sound signal, the data on the EDA indicators of all subjects under the S2 experiment condition were age-differentiated (Junior, 3–4 years, Middle, 4–5 years and Senior, 5–6 years), and a paired *t*-test of 95% confidence levels was used to verify the hypothesis. As shown in Table 6, *p* < 0.05 held true for the differences among different age groups in all physiological indicators except SCP, indicating that age significantly affected children’s risk perception.

Table 7 shows the scores of EDA indicators and their relative change from the baseline to the S2 experiment. It can be seen that the SC and SCT scores during the S2 experiment were significantly higher than those at baseline. The order of the relative change of SC in different age groups is as follows: Senior > Middle > Junior. Specifically, the relative change of SC was 11.35% greater in the middle class than in the junior classes, and 14.90% greater in the senior class than in the middle class. Similarly, the order of the relative change of SCT in different age groups is as follows: Senior > Middle > Junior, with the relative change of SCT being 11.48% greater in the senior class than in the middle class and 13.88% greater in the middle class than in the junior class. These results indicate that the older the child, the higher the conductivity of the skin under the action of the alarm sound. In other words, from the perspective of EDA indicators, as children age, they become more sensitive to the evacuation sound signal. This is consistent with the results of previous research on the evacuation dynamics of kindergarten children that the older the children are, the shorter the evacuation pre-action time and the quicker the evacuation [2,5,13]. However, this study has considered changes in children’s physiological indicators and analysed this phenomenon on a more fundamental level.

## 4. Discussion and Limitation

This study provides new venues for a better understanding of evacuation behaviours and may have a large impact on fire safety research and management. This study presents the potential of physiological sensory data collected from wearable sensors to understand children’s perceived risk during evacuation processes, by showing differences in EDA and HRV between different alarm sound conditions and the effects of gender and age. Children’s EDA during the warning alarm was significantly higher than EDA during the message alert condition. However, there were no statistically significant differences in the score of SCP, ULF and VLF between different sound types. Considering that SCP is a phasic component of EDA that reflects short-term responses to external stimulus and evacuation of children is a dynamic process in emergency situations, SCP may be affected by finger movement. Therefore, SC, SCT, and SCL would be more related to risk perception than SCP. On the other hand, ULF and VLF do not reflect by alarm sounds. A possible reason is the frequency of ULF and VLF is very low. Children’s heartbeat is not as strong as adults, so they cannot be affected by experiments. Compare to the current survey-based methods to measure occupants’ perceived risk, physiological response acquired from wearable sensors will allow us to develop objective and real-time mechanisms for understanding occupants’ perceived risk.

Physiological responses collected from wearable devices are expected to deepen our understanding of risk perception during emergency situations. Since wearable devices measure an individual’s physiological response to hazard, it would be more related to one’s own perceived risk. This personalized measurement could offer a strong foundation to explore new areas for risk perception studies, even for risk assessment. For example, the perceived risk estimated from physiological responses can be integrated with other reliable risk assessment methods such as total efficiency risk priority number (TERPN) [61].

This study presents children are more sensitive to the warning alarm, which is different from the results of previous studies of evacuation alarms in adults [16,18,19,20]. In the past, there has been no research on the effects of different types of alarm sounds on children’s evacuation in public spaces like kindergartens. Now, we have used a scientific and objective measurement method (physiological indicators) to identify which types of alarm sounds are better for children risk perception, this could be very beneficial for children’s evacuation and safety, and may have a large impact on preschool safety management.

Although this study successfully demonstrated the feasibility of using physiological sensory data acquired from wearable sensors to understand children’s perceived risk during they exposure to different alarm sounds and investigated the optimum alarm for children, several limitations should be acknowledged. First, only 42 people were divided into six groups to participate in this study. In the future, we will take a greater number of participants to understand sympathetic arousal caused by risk and other occupants. Second, only physiological indicators of EDA and HRV were studied and analysed in this study. Other relevant physiological factors, such as the electrical activity of the brain measured by electroencephalography (EEG), will be the subject of future research.

## 5. Conclusions

This paper proposes a novel method to obtain physiological indicator data through wearable sensors to explore the changes in children’s physiological responses to different types of alarm sounds in emergencies by evaluating the effects of different alarm sound types on children’s risk perception. In this study, children’s physiological responses were found to be strongly influenced by the type of alarm sound they heard. In order to achieve the research goal, on-site evacuation experiments were conducted on 42 children aged 3–6 years, using three types of alarm sounds. Through data analysis, the following conclusions can be drawn:

The alarm sound can trigger changes in physiological indicators in children aged 3–6 years. Different alarm sound types have significantly different effects on EDA and HRV (*p* < 0.05). In addition, SC, SCT and SCL can be used as the main indicators for analysing EDA of children (*p* < 0.05), and ULF and VLF (*p* > 0.05) for analysing HRV are not affected by the type of alarm sound in children aged 3–6 years.

Kindergarten children are more sensitive to the sound of warning alarm signals than voice messages, and the combined voice alert and warning alarm had the strongest effect in stimulating the children to perceive risks. This is significantly different from previous studies of adults showing them to be more sensitive to voice alert messages. In terms of voice messages, children are more sensitive to short keywords than complete sentences, because children aged 3–6 years have not developed good feedback to language.

Gender has a significant impact on children’s evacuation sound signal reception, and girls are more sensitive than boys to evacuation sound signals. This result is basically consistent with the results of studies of risk perception in adults. Whether the difference in sensitivity to risks between different genders is inherent requires further discussion in the future.

For children aged 3–6 years, age has a significant impact on children’s evacuation sound signal reception. The older the child, the more sensitive he/she is to the alarm sound signal, and the stronger the risk awareness. This is consistent with the results of previous research on the evacuation dynamics of kindergarten children; the older the children, the shorter their evacuation pre-action time, and the quicker their evacuation. This study considered changes in children’s physiological indicators and explained the phenomenon in a more fundamental sense.

## Figures and Tables

**Table 1:** Information on the participating children.

Class	Age (years)	Number	Male	Female
Junior	3–4	14	7	7
Middle	4–5	14	8	6
Senior	5–6	14	8	6

**Table 2:** Information on the alarm sound types.

Type	S1	S2	S3
LAeq (dB)	88.13 dB	87.32 dB	88.11dB
Description	Voice alert (loop play of Chinese words: “Fire, run”)	Warning alarm (commonly used alarm in fire drills)	Combined voice alert and warning alarm (Chinese voice alert is inserted in warning alarm)
Alarm duration:	From the beginning of the experiment, until children are completely evacuated from the activity room		
Duration between each of the alarm type (min)	10 min		
LAeq is the A-weighted equivalent continuous sound level in decibels measured over a stated period of time			

**Table 3:** Average scores of physiological indicators.

Physiological Indicators		Baseline	S1	S2	S3
		Mean	Mean	Mean	Mean
EDA	SC (μS)	8.43	11.92	12.33	14.36
	SCT (μS)	7.60	9.36	9.84	11.55
	SCP (μS)	0.82	2.56	2.49	2.81
	LATENCY (s)		1.97	1.97	1.96
	AMP (μS)		1.94	1.75	1.93
	SCL (μS)		7.96	9.46	9.79
HRV	IBI (ms)	607.89	613.07	637.05	634.78
	SDNN (ms)	59.77	108.70	139.12	148.71
	RMSSD (ms)	63.74	127.25	184.63	177.63
	SDSD (ms)	63.93	129.92	189.37	181.94
	ULF (ms²)	45.10	50.55	51.03	69.94
	VLF (ms²)	596.22	727.55	856.21	1034.14
	LF (ms²)	1288.74	3612.94	4318.82	4637.84
	HF (ms²)	1132.01	4673.58	7220.49	8331.11
	LF/HF	1.89	1.71	0.70	1.00

EDA: Electrodermal activity, HRV: Heart rate variability, SC: Skin conductivity, SCT: Skin conductive tonic, SCP: Skin conductance phasic, LATENCY: latency for each event stimulus, AMP: Amplitude, SCL: Skin conductance level, IBI: Inter-beat interval, SDNN: Standard deviation of NN intervals, RMSSD: The square root of the mean of the sum of the squares of differences between adjacent NN intervals, SDSD: Standard deviation of standard deviation of NN intervals, ULF: Ultra-low frequency, VLF: Very low frequency, LF: Low frequency, HF: High frequency.

**Table 4:** Paired *t*-tests between experiments of assessment with objective measures.

Physiological Indicators		S1-Baseline	S2-Baseline	S3-Baseline	S1-S2-S3
		Sig.	Sig.	Sig.	Sig.
EDA	SC (μS)	0.000	0.000	0.000	0.000
	SCT (μS)	0.003	0.001	0.000	0.000
	SCP (μS)	0.000	0.000	0.000	0.060
	LATENCY (s)				0.996
	AMP (μS)				0.752
	SCL (μS)				0.000
HRV	IBI (ms)	0.652	0.021	0.021	0.000
	ULF (ms²)	0.626	0.641	0.054	0.000
	VLF (ms²)	0.263	0.079	0.025	0.000
	LF (ms²)	0.000	0.000	0.000	0.000
	HF (ms²)	0.000	0.000	0.000	0.000
	LF/HF	0.703	0.000	0.005	0.000

Sig.: significance is *p* value.

**Table 5:** Scores of baseline and S2 of physiological indicators.

Physiological Indicators	Baseline (Mean)		S2 (Mean)					
	Male	Female	Male	△Male	Relative Change M (%)	Female	△Female	Relative Change F (%)
SC	8.16	8.71	11.18	3.02	37.00	13.53	4.82	55.33
SCT	7.29	7.93	8.87	1.58	21.67	10.86	2.93	36.94
SCP	0.87	0.77	2.31	1.44	165.51	2.67	1.9	246.75
SCL			7.78			10.85		
IBI	603.2	612.82	624.6	21.4	3.54	650.11	37.29	6.08
SDNN	61.12	58.36	131.14	70.02	114.56	147.49	89.13	152.72
RMSSD	68.79	58.44	177.81	109.02	158.48	191.79	133.35	228.18
LF	1419.52	1151.43	3611	2191.48	154.38	5062.04	3910.61	339.63
HF	1351.72	901.31	6313.2	4961.48	367.04	8173.14	7271.83	806.80
LF/HF	1.74	2.05	0.76	−0.98	−56.32	0.64	−1.41	−68.78

**△:** alarm sounds test value − baseline value.

**Table 6:** Paired *t*-test between age and S2.

Physiological Indicators	*t*	Sig.
SC	−8.546	0.000
SCT	−7.918	0.000
SCP	−1.299	0.201

Sig.: significance is *p* value.

**Table 7:** Scores of baseline and S2 of physiological indicators.

Physiological Indicators	Baseline			S2						Relative Change		
	Junior	Middle	Senior	Junior	△Junior	Middle	△Middle	Senior	△Senior	Junior (%)	Middle (%)	Senior (%)
SC	9.43	7.23	8.54	12.65	3.22	10.51	3.29	13.69	5.15	34.12	45.47	60.37
SCT	8.17	6.64	7.93	9.52	1.35	8.66	2.02	11.25	3.32	16.51	30.39	41.87
SCP	1.26	0.58	0.61	3.13	1.87	1.85	1.27	2.44	1.83	48.20	217.40	302.15

**△**: alarm sounds test value − baseline value.

## References

[1] Mytton J., Goodenough T., Novak C. (2017). Children and young people’s behaviour in accidental dwelling fires: A systematic review of the qualitative literature. Saf. Sci..

[2] Kholshevnikov V.V., Samoshin D.A., Parfenenko A.P. Pre-school and school children building evacuation. Proceedings of the Fourth International Symposium on Human Behaviour in Fire 2009.

[3] Kholshchevnikov V.V., Samoshin D.A., Parfyonenko A.P., Belosokhov I.P. (2012). Study of children evacuation from pre-school education institutions. Fire Mater..

[4] Cuesta A., Gwynne S.M.V. (2016). The collection and compilation of school evacuation data for model use. Saf. Sci..

[5] Fang Z.M., Jiang L.X., Li X.L., Qi W., Chen L.Z. (2019). Experimental study on the movement characteristics of 5–6 years old Chinese children when egressing from a pre-school building. Saf. Sci..

[6] Chu H., Yu J., Wen J., Yi M., Chen Y. (2019). Emergency Evacuation Simulation and Management Optimization in Urban Residential Communities. Sustainability.

[7] van der Wal C.N., Formolo D., Robinson M.A., Minkov M., Bosse T. (2017). Simulating crowd evacuation with socio-cultural, cognitive, and emotional elements. Transactions on Computational Collective Intelligence XXVII.

[8] Larusdottir A.R., Dederichs A.S. (2011). A step towards including children’s evacuation parameters and behavior in fire safe building design. Fire Saf. Sci..

[9] Larusdottir A.R., Dederichs A.S. (2011). Evacuation dynamics of children–walking speeds, flows through doors in daycare centers. Pedestrian and Evacuation Dynamics.

[10] Larusdottir A.R., Dederichs A.S. (2012). Evacuation of children: Movement on stairs and on horizontal plane. Fire Technol..

[11] Li H., Zhang J., Yang L.,  Song W., Yuen K.K.R. (2020). A comparative study on the bottleneck flow between preschool children and adults under different movement motivations. Saf. Sci..

[12] Hamilton G.N., Lennon P.F., O’Raw J. (2017). Human behaviour during evacuation of primary schools: Investigations on pre-evacuation times, movement on stairways and movement on the horizontal plane. Fire Saf. J..

[13] Najmanová H., Ronchi E. (2017). An experimental data-set on pre-school children evacuation. Fire Technol..

[14] Wu Y., Kang J., Wang C. (2018). A crowd route choice evacuation model in large indoor building spaces. Front. Archit. Res..

[15] Purser D.A., Bensilum M. (2001). Quantification of behaviour for engineering design standards and escape time calculations. Saf. Sci..

[16] Purser D. (2008). Comparisons of evacuation efficiency and pre-travel activity times in response to a sounder and two different voice alarm messages. Pedestrian and Evacuation Dynamics 2010.

[17] Fire D.B.S.B.D. (1997). Safety Engineering in Buildings. Part 1: Guide to the Application of Fire Safety Engineering Principles.

[18] Bellamy L.L., Geyer T.A.W. (1990). Experimental Programme to Investigate Informative Fire Warning Characteristics for Motivating Fast Evacuation.

[19] Cable E.A. Cry wolf syndrome: Radical changes solve the false alarm problem. Proceedings of the Fouth National Symposium Trade Exhibition on Health Care Safety and the Enviornment.

[20] Proulx G., Sime J.D. (1991). To prevent’ panic’ in an underground emergency: Why not tell people the truth?. Fire Saf. Sci..

[21] Bryan J.L. (1957). A Study of the Survivors Reports on the Panic in the Fire at the Arundel Park Hall in Brooklyn, Maryland on 29 January 1956.

[22] Baek C.H., Park S.M., Choi B.J. (2019). Analysis of the Relationship between Human Risk Factors and Evacuation Behavior for Tunnel Safety. J. Korea Acad. Ind. Coop. Soc..

[23] Tong D., Canter D. (1985). The decision to evacuate: A study of the motivations which contribute to evacuation in the event of fire. Fire Saf. J..

[24] Urbina E., Wolshon B. (2003). National review of hurricane evacuation plans and policies: A comparison and contrast of state practices. Transp. Res. Part A Policy Pract..

[25] Montz B.E., Tobin G.A., Hagelman R.R. (2017). Natural Hazards: Explanation and Integration.

[26] Kinateder M., Ronchi E., Nilsson D., Kobes M., Müller M., Pauli P., Mühlberger A. Virtual reality for fire evacuation research. Proceedings of the 2014 Federated Conference on Computer Science and Information Systems.

[27] Zou H., Li N., Cao L. (2017). Emotional response–based approach for assessing the sense of presence of subjects in virtual building evacuation studies. J. Comput. Civ. Eng..

[28] Medvedev O., Shepherd D., Hautus M.J. (2015). The restorative potential of soundscapes: A physiological investigation. Appl. Acoust..

[29] Chuen L., Sears D., McAdams S. (2016). Psychophysiological responses to auditory change. Psychophysiology.

[30] Kang J., Aletta F., Gjestland T.T., Brown L.A., Botteldooren D., Schulte-Fortkamp B., Coelho J.L.B. (2016). Ten questions on the soundscapes of the built environment. Build. Environ..

[31] Li Z., Kang J. (2019). Sensitivity analysis of changes in human physiological indicators observed in soundscapes. Landsc. Urban Plan..

[32] Wilson G.M., Sasse M.A. (2000). Do Users Always Know What’s Good for Them? Utilising Physiological Responses to Assess Media Quality. People and Computers XIV—Usability or Else.

[33] Ravaja N., Kallinen K., Saari T., Keltikangas-Jarvinen L. (2004). Suboptimal exposure to facial expressions when viewing video messages from a small screen: Effects on emotion, attention, and memory. J. Exp. Psychol. Appl..

[34] Doorley R., Pakrashi V., Byrne E., Comerford S., Ghosh B., Groeger J.A. (2015). Analysis of heart rate variability amongst cyclists under perceived variations of risk exposure. Transp. Res. Part F Traffic Psychol. Behav..

[35] Appelhans B.M., Luecken L.J. (2006). Heart rate variability as an index of regulated emotional responding. Rev. Gen. Psychol..

[36] Dishman R.K., Nakamura Y., Garcia M.E., Thompson R.W., Blair S.N. (2000). Heart rate variability, trait anxiety, and perceived stress among physically fit men and women. Int. J. Psychophysiol..

[37] Choi B., Jebelli H., Lee S. (2019). Feasibility analysis of electrodermal activity EDA acquired from wearable sensors to assess construction workers’ perceived risk. Saf. Sci..

[38] Suied C., Susini P., Mcadams S. (2008). Evaluating warning sound urgency with reaction times. J. Exp. Psychol. Appl..

[39] Houtkamp J.M., Toet A., Bos F.A. (2012). Task-relevant sound and user experience in computer-mediated firefighter training. Simul. Gaming.

[40] Allanson J., Fairclough S.H. (2004). A research agenda for physiological computing. Interact. Comput..

[41] Kennedy D.K., Hughes B.M. (2004). The optimism-neuroticism question: An evaluation based on cardiovascular reactivity in female college students. Psychol. Rec..

[42] Erfanian M., Mitchell A.J., Kang J., Aletta F. (2019). The Psychophysiological Implications of Soundscape: A Systematic Review of Empirical Literature and a Research Agenda. Int. J. Environ. Res. Public Health.

[43] Meehan M., Razzaque S., Insko B., Whitton M., Brooks F.P. (2005). Review of four studies on the use of physiological reaction as a measure of presence in stressful virtual environments. Appl. Psychophysiol. Biofeedback.

[44] Picard R.W., Fedor S., Ayzenberg Y. (2016). Multiple arousal theory and daily-life electrodermal activity asymmetry. Emot. Rev..

[45] Fowles D.C., Kochanska G., Murray K. (2000). Electrodermal activity and temperament in preschool children. Psychophysiology.

[46] The Effects of A Pressure Vest on Task Engagement, Challenging Behavior, and a Physiological Measure of Stress for a Child with Intellectual Disability. http://hdl.handle.net/2142/79051.

[47] Lindquist M., Lange E., Kang J. (2016). From 3D landscape visualization to environmental simulation: The contribution of sound to the perception of virtual environments. Landsc. Urban Plan..

[48] Cadena L.F.H., Soares A.C.L., Pavón I., Coelho L.B. (2017). Assessing soundscape: Comparison between in situ and laboratory methodologies. Noise Mapp..

[49] Alvarsson J.J., Wiens S., Nilsson M.E. (2010). Stress recovery during exposure to nature sound and environmental noise. Int. J. Environ. Res. Public Health.

[50] Kang J., Zhang M. (2010). Semantic differential analysis of the soundscape in urban open public spaces. Build. Environ..

[51] Ba M., Kang J. (2019). Effect of a fragrant tree on the perception of traffic noise. Build. Environ..

[52] Fuller B.F. (1992). The effects of stress-anxiety and coping styles on heart rate variability. Int. J. Psychophysiol..

[53] Fabes R.A., Eisenberg N., Eisenbud L. (1993). Behavioral and physiological correlates of children’s reactions to others in distress. Dev. Psychol..

[54] Gorman J.M., Sloan R.P. (2000). Heart rate variability in depressive and anxiety disorders. Am. Heart J..

[55] Sgoifo A., Braglia F., Costoli T., Musso E., Meerlo P., Ceresini G., Troisi A. (2003). Cardiac autonomic reactivity and salivary cortisol in men and women exposed to social stressors: Relationship with individual ethological profile. Neurosci. Biobehav. Rev..

[56] Murakami H., Ohira H. (2007). Influence of attention manipulation on emotion and autonomic responses. Percept. Mot. Skills.

[57] Hume K., Ahtamad M. (2013). Physiological responses to and subjective estimates of soundscape elements. Appl. Acoust..

[58] Proulx G. (1995). Evacuation time and movement in apartment buildings. Fire Saf. J..

[59] Rundmo T. (1999). Perceived risk, health and consumer behaviour. J. Risk Res..

[60] Drottz B.M. (1991). Perception of Risk: Studies of Risk Attitudes, Perceptions and Definitions.

[61] Di Bona G., Silvestri A., Forcina A., Petrillo A. (2018). Total efficient risk priority number TERPN: A new method for risk assessment. J. Risk Res..

